# Supplementary material for: Serum IgA and IgM levels in hemochromatosis probands with HFE p.C282Y homozygosity
Source: PLoS One. 2026 Jun 24;21(6):e0352148. doi: 10.1371/journal.pone.0352148 (PMC13293435; doi:10.1371/journal.pone.0352148)
Supplement: S1 Table — (PDF) [file pone.0352148.s001.pdf]

Supporting information

Supplemental Tables 1-5: Serum IgA and IgM levels of European adults

Serum IgA and IgM levels in hemochromatosis probands with *HFE* p.C282Y homozygosity

Abbreviated title: Serum IgA and IgM in hemochromatosis

James C. Barton[1,2,3*], J. Clayborn Barton[2], Luigi F. Bertoli[2,3], and Ronald T. Acton[2,4]

[1] Department of Medicine, University of Alabama at Birmingham, Birmingham, Alabama, USA

[2] Southern Iron Disorders Center, Birmingham, Alabama, USA

[3] Department of Medicine, Brookwood Baptist Medical Center, Birmingham, Alabama, USA

[4] Department of Microbiology, University of Alabama at Birmingham, Birmingham, Alabama, USA

*Corresponding author

E-mail: bartonjames336@gmail.com

Introduction

We sought to compare the mean serum immunoglobulin A (IgA) and immunoglobulin M (IgM) levels of a cohort of 73 referred Alabama adults with hemochromatosis and *HFE* p.C282Y homozygosity with those of published cohorts of European adults not selected for hemochromatosis diagnoses or *HFE* genotypes.

Methods

We performed computerized and manual searches to identify a convenience sample of published reports of serum IgA and IgM measured in cohorts of more than 50 healthy, "control," or general population European adults aged ≥18 y not selected for hemochromatosis diagnoses or *HFE* genotypes. We selected reports that described laboratory methodology for measurement of serum IgA and IgM and expressed cohort results as mean, standard deviation (SD), and 95% confidence interval (CI). We converted IgA and IgM measures published as IU/L to g/L according to the method of Rowe et al. [1]. We excluded reports in which serum IgA and IgM measurements were expressed as medians.

We used a Cochrane's formula [2] to combine the Ig-specific n, mean, and SD from multiple published cohorts into descriptors of a single group. We used a two-sample t test and n, mean, and SD from each group to compare Alabama proband data with those of Europeans not selected for hemochromatosis diagnoses or *HFE* genotype. We defined values of $p < 0.05$ as significant.

Results

Supplemental Table 1. Serum IgA and IgM levels of adults

**Author (year)[a]**	**Reference**	**Adults, n**	**Method**	**Mean IgA ± SD, g/L [95% CI]**	**Mean IgM ± SD, g/L [95% CI]**
Šinkov (1973)	[3]	60	radial immunodiffusion	1.62 ± 0.58 [0.93, 2.75]	1.11 ± 0.39 [0.35, 1.87]
Veys (1973)	[4]	296	"linear plate" immunodiffusion	2.08 ± 1.81 [0.79, 6.31]	0.74 ± 0.87 [0.30, 2.00]
Ghessi (1976)	[5]	603	single radial immunodiffusion	2.36 ± 0.74 [0.91, 3.81]	n.a.
Quintiliani (1976)	[6]	773	immunodiffusion	1.83 ± 0.65 [0.55, 3.11]	1.50 ± 0.65 [0.22, 2.78]
Gonzalez-Quintela (2008)	[7]	460	chemiluminescent enzyme immunoassay	2.62 ± 1.19 [0.87, 5.76]	1.47 ± 0.84 [0.46, 3.86]
Puissant-Lubrano (2015)	[8]	270	immunoturbidimetry	2.12 ± 0.77 [0.91, 3.93]	n.a.
Combined European data	*[3-8]*	*2462 (IgA); 1589 (IgM)*	-	*2.16 ± 1.04 [2.12, 2.20]*	*1.34 ± 0.80 [1.30, 1.38]*
Present cohort (2025)	-	73	nephelometry	2.11 ± 1.06 [1.87, 2.35]	1.11 ± 0.75 [0.94, 1.28]
				p = 0.686	p = 0.015

CI, confidence interval; n.a., not available; SD, standard deviation.

[a] Šinkov: healthy individuals aged 21-50 y from Sofia, Bulgaria (30 men, 30 women); Veys: employees aged 20-65 y of the Post, Telegraph and Telephone Administration, Ghent, Belgium ("P.T.T. new"; 296 employees (numbers of men and women not specified)); Ghessi: 603 healthy blood donors aged 21-65 y from Lombardy, Italy (510 men, 93 women); Quintiliani: healthy blood donors aged 20-59 y in Rome, Italy (408 men, 365 women); Gonzalez-Quintela: Caucasian adults aged 18-92 y (median 54 y) from the general population of A-Estrada, northwestern Spain (203 men, 44.1% men); Puissant-Lubrano: 270 healthy blood donors aged 18-68 y from Toulouse, France (136 men, 134

women); Present cohort: referred hemochromatosis probands with *HFE* p.C282Y homozygosity aged 22-80 y (mean 51 ± 13 y) from Alabama, USA (36 men, 37 women).

Supplemental Table 2. Serum IgA levels of men[a]

**Author (year)**[a]	**Reference**	**n**	**Method**	**Mean IgA ± SD, g/L [95% CI]**
Ghessi (1976)	[5]	510	single radial immunodiffusion	2.38 ± 0.73 [0.91, 3.84]
Quintiliani (1976)	[6]	408	immunodiffusion	1.86 ± 0.69 [0.51, 3.21]
Combined European data	*[5,6]*	*918*	-	*2.15 ± 0.76 [2.10, 2.20]*
Present cohort (2025)	-	36	nephelometry	2.19 ± 0.91 [1.89, 2.49]
				p = 0.759

CI, confidence interval; SD, standard deviation.

[a] Ghessi: healthy blood donors aged 21-65 y from Lombardy, Italy; Quintiliani: healthy blood donors aged 20-59 y in Rome, Italy; Present cohort: referred hemochromatosis probands with *HFE* p.C282Y homozygosity aged 22-80 y (mean 50 ± 13 y) from Alabama, USA. CI, confidence interval.

Supplemental Table 3. Serum IgA levels of women[a]

**Author (year)**[a]	**Reference**	**n**	**Method**	**Mean IgA ± SD, g/L [95% CI]**
Ghessi (1976)	[5]	93	single radial immunodiffusion	2.23 ± 0.75 [0.73, 3.74]
Quintiliani (1976)	[6]	365	immunodiffusion	1.80 ± 0.60 [0.60, 3.00]
Combined European data	*[5,6]*	*458*	-	*1.89 ± 0.66 [1.83, 1.95]*
Present cohort (2025)	-	37	nephelometry	2.04 ± 1.20 [1.65, 2.43]
				p = 0.219

CI, confidence interval; SD, standard deviation.

[a] Ghessi: healthy blood donors aged 21-65 y from Lombardy, Italy; Quintiliani: healthy blood donors aged 20-59 y in Rome, Italy; Present cohort: referred hemochromatosis probands with *HFE* p.C282Y homozygosity aged 22-80 y (mean 50 ± 13 y) from Alabama, USA. CI, confidence interval.

Supplemental Table 4. Serum IgM levels of men[a]

Author (year)	Reference	n	Method	Mean IgM ± SD, g/L [95% CI]
Šinkov (1973)	[3]	30	radial immunodiffusion	0.97 ± 0.44 [0.12, 1.83]
Ghessi (1976)	[5]	510	single radial immunodiffusion	1.50 ± 0.48 [0.57, 3.94]
Quintiliani (1976)	[6]	408	immunodiffusion	1.33 ± 0.57 [0.26, 2.44]
Puissant-Lubrano (2015)	[8]	136	immunoturbidimetry	0.90 ± 0.49 [0.32, 2.23]
Combined European data	*[3,5,6,8]*	*1084*	-	*1.35 ± 0.55 [1.32, 1.38]*
Present cohort (2025)	-	36	nephelometry	1.03 ± 0.84 [0.76, 1.30]
				p <0.001

CI, confidence interval; SD, standard deviation.

[a] Šinkov: healthy individuals aged 21-50 y from Sofia, Bulgaria; Ghessi: healthy blood donors aged 21-65 y from Lombardy, Italy; Quintiliani: healthy blood donors aged 20-59 y in Rome, Italy; Puissant-Lubrano: healthy blood donors aged 18-68 y from Toulouse, France; Present cohort: referred hemochromatosis probands with *HFE* p.C282Y homozygosity aged 22-80 y (mean 50 ± 13 y) from Alabama, USA.

Supplemental Table 5. Serum IgM levels of women[a]

**Author (year)**[a]	**Reference**	**n**	**Method**	**Mean IgM ± SD, g/L [95% CI]**
Šinkov (1973)	[3]	30	radial immunodiffusion	1.25 ± 0.29 [0.9, 1.82]
Ghessi (1976)	[5]	93	single radial immunodiffusion	1.89 ± 0.49 [0.71, 5.05]
Quintiliani (1976)	[6]	365	immunodiffusion	1.69 ± 0.69 [0.33, 3.04]
Puissant-Lubrano (2015)	[8]	134	immunoturbidimetry	1.10 ± 0.54 [0.50, 2.60]
Combined European data	*[3,5,6,8]*	*622*	-	*1.57 ± 0.68 (1.52, 1.62)*
Present cohort (2025)	-	37	nephelometry	1.18 ± 0.67 [0.96, 1.40]
				$p < 0.001$

CI, confidence interval; SD, standard deviation.

[a] Šinkov: healthy individuals aged 21-50 y from Sofia, Bulgaria; Ghessi: healthy blood donors aged 21-65 y from Lombardy, Italy; Quintiliani: healthy blood donors aged 20-59 y in Rome, Italy; Puissant-Lubrano: healthy blood donors aged 18-68 y from Toulouse, France; Present cohort: referred hemochromatosis probands with *HFE* p.C282Y homozygosity aged 22-80 y (mean 50 ± 13 y) from Alabama, USA.

References for Supporting Tables

1. Rowe DS, Grab B, Anderson SG: An international reference preparation for human serum immunoglobulins G, A and M: content of immunoglobulins by weight. Bull World Health Organ. 1972, 46:67-79.

2. Higgins JPT, Li T, Deeks JJ: Choosing effect measures and computing estimates of effect. Cochrane Handbook for Systematic Reviews of Interventions version 6.5. Higgins JPT, Thomas J, Chandler J, et al. (eds): Cochrane, London; 2024.

3. Šinkov D, Tolev V, Štereva T: Concentration of IgG, IgA, and IgM in terms of international units in the sera of healthy individuals. Bull World Health Organ. 1973, 49:217-8.

4. Veys EM, Wieme RJ: Serum IgG, IgM and IgA concentration determined by the "linear plate" immunodiffusion technique in a normal population. Clin Chim Acta. 1973, 47:295-306. 10.1016/0009-8981(73)90327-6

5. Ghessi A, Azzario F, Marinig C, et al.: [IgA-IgG-IgM serum levels in blood donors. Examination of some variables]. Boll Ist Sieroter Milan. 1976, 55:225-33.

6. Quintiliani L, Taggi F, Giuliani E, et al.: IgG, IgA and IgM concentration in human sera from different age groups: statistical evaluation. Boll Ist Sieroter Milan. 1976, 55:241-8.

7. Gonzalez-Quintela A, Alende R, Gude F, et al.: Serum levels of immunoglobulins (IgG, IgA, IgM) in a general adult population and their relationship with alcohol consumption, smoking and common metabolic abnormalities. Clin Exp Immunol. 2008, 151:42-50. 10.1111/j.1365-2249.2007.03545.x

8. Puissant-Lubrano B, Peres M, Apoil PA, et al.: Immunoglobulin IgA, IgD, IgG, IgM and IgG subclass reference values in adults. Clin Chem Lab Med. 2015, 53:e359-e361. 10.1515/cclm-2014-1186
